# Larval Development of *Aedes aegypti* and *Aedes albopictus* in Peri-Urban Brackish Water and Its Implications for Transmission of Arboviral Diseases

**DOI:** 10.1371/journal.pntd.0001369

**Published:** 2011-11-22

**Authors:** Ranjan Ramasamy, Sinnathamby N. Surendran, Pavilupillai J. Jude, Sangaralingam Dharshini, Muthuladchumy Vinobaba

**Affiliations:** 1 Institute of Health Sciences, Universiti Brunei Darussalam, Gadong, Brunei Darussalam; 2 Department of Zoology, Faculty of Science, University of Jaffna, Jaffna, Sri Lanka; 3 Department of Zoology, Faculty of Science, Eastern University, Chenkaladi, Sri Lanka; Centers for Disease Control and Prevention, Puerto Rico, United States of America

## Abstract

*Aedes aegypti* (Linnaeus) and *Aedes albopictus* Skuse mosquitoes transmit serious human arboviral diseases including yellow fever, dengue and chikungunya in many tropical and sub-tropical countries. Females of the two species have adapted to undergo preimaginal development in natural or artificial collections of freshwater near human habitations and feed on human blood. While there is an effective vaccine against yellow fever, the control of dengue and chikungunya is mainly dependent on reducing freshwater preimaginal development habitats of the two vectors. We show here that *Ae. aegypti* and *Ae. albopictus* lay eggs and their larvae survive to emerge as adults in brackish water (water with <0.5 ppt or parts per thousand, 0.5–30 ppt and >30 ppt salt are termed fresh, brackish and saline respectively). Brackish water with salinity of 2 to 15 ppt in discarded plastic and glass containers, abandoned fishing boats and unused wells in coastal peri-urban environment were found to contain *Ae. aegypti* and *Ae. albopictus* larvae. Relatively high incidence of dengue in Jaffna city, Sri Lanka was observed in the vicinity of brackish water habitats containing *Ae. aegypti* larvae. These observations raise the possibility that brackish water-adapted *Ae. aegypti* and *Ae. albopictus* may play a hitherto unrecognized role in transmitting dengue, chikungunya and yellow fever in coastal urban areas. National and international health authorities therefore need to take the findings into consideration and extend their vector control efforts, which are presently focused on urban freshwater habitats, to include brackish water larval development habitats.

## Author Summary

*Aedes aegypti* and *Aedes albopictus* mosquitoes transmit arboviral disease like dengue and chikungunya that are of international concern. Control of dengue and chikungunya presently focuses on eliminating freshwater larval development habitats of the two mosquitoes in urban surroundings. We investigated the ability of the two mosquito species to lay eggs and undergo development into larvae, pupae and adults in brackish water, and examined brackish water collections in the peri-urban environment for the presence of larvae. The results confirmed their ability to lay eggs and for the eggs to develop into adults in brackish water. Their larvae were found in brackish water in discarded food/beverage containers and abandoned boats as well as disused wells. Such brackish water collections with larvae in Jaffna city, Sri Lanka were found near areas of high dengue incidence. This hitherto unappreciated potential contribution to arboviral disease transmission in urban areas is of global significance. National and international health authorities need to take these new findings into consideration in developing appropriate strategies for controlling diseases transmitted by the two mosquito species.

## Introduction

*Aedes aegypti* (Linnaeus) (Diptera: Culicidae) is the principal tropical mosquito vector of arboviruses causing yellow fever, dengue and chikungunya [1]–[3]. The related *Aedes albopictus* Skuse is a secondary vector of dengue and chikungunya [1]–[4]. Unlike *Ae. aegypti*, *Ae. albopictus* possesses a diapausing egg stage to survive winters which has enabled it to spread to temperate regions and cause a chikungunya epidemic in northern Italy in 2007 [4].

Dengue is the most common arboviral disease of humans, with 50 million annual cases in more than 100 countries, an increasing incidence and spread worldwide, and 2.5 billion people at risk [5], [6]. About 500,000 persons require hospitalization every year for dengue hemorrhagic fever (DHF) and 2.5% of DHF cases are fatal [6]. Dengue is endemic in Sri Lanka with a high incidence in the northern and eastern districts of Jaffna and Batticaloa respectively [7]. Sri Lanka moreover experienced an epidemic of chikungunya in 2006–2007 [8]. Dengue is also endemic in Brunei and many other Southeast Asian countries [5], [9], [10]. There is presently no licensed vaccine or specific anti-viral drug for dengue [6], [9].Yellow fever, another flaviviral disease, is endemic in Africa and South America, has a zoonotic reservoir and is responsible for 200,000 cases and 30,000 deaths worldwide [11]. An effective vaccine is available against yellow fever, but it can potentially spread to Asia through increased global transport. Chikungunya, caused by an alphavirus, is endemic in Southeast Asia and has produced recent epidemics in Africa and South Asia [1], [2], [12]. Additional arboviral diseases with animal reservoirs are emerging as serious threats to human health [1], [13].

*Ae. aegypti* and *Ae. albopictus* have adapted to feed on humans and undergo larval and pupal development in natural ( e.g. rock pools, tree holes, leaf axils) and artificial (e.g. water tanks, blocked drains, decorative pots and discarded tyres and food/beverage containers) freshwater collections in the urban and peri-urban environment [1], [3], [5]. *Ae. aegypti* and *Ae. albopictus* are common anthropophagic mosquitoes in urban areas of Sri Lanka [14], [15]. Control of dengue and chikungunya in tropical countries is mainly achieved through surveillance for *Ae. aegypti* and *Ae. albopictus* larvae and eliminating larval development habitats, with the attendant use of insecticides and public education [5], [9], [10]. Larval control efforts invariably focus on freshwater development habitats of the two mosquito species near human habitations [5], [9], [10]. We investigated a hypothesis that *Ae. aegypti* and *Ae. albopictus* also undergo preimaginal development in brackish water in the peri-urban environment (water with <0.5 ppt or parts per thousand, 0.5–30 ppt and >30 ppt salt are termed fresh, brackish and saline respectively), with the potential thereby to make a hitherto unrecognized contribution to the transmission of dengue, chikungunya and other arboviral diseases.

## Methods

### Ethics statement

Head of each household was explained the purpose and the objectives of the study and his/her informed consent was obtained orally to inspect wells for the presence of *Aedes* larvae and to measure the salinity of well water.

### Study sites

**Figure 1:**
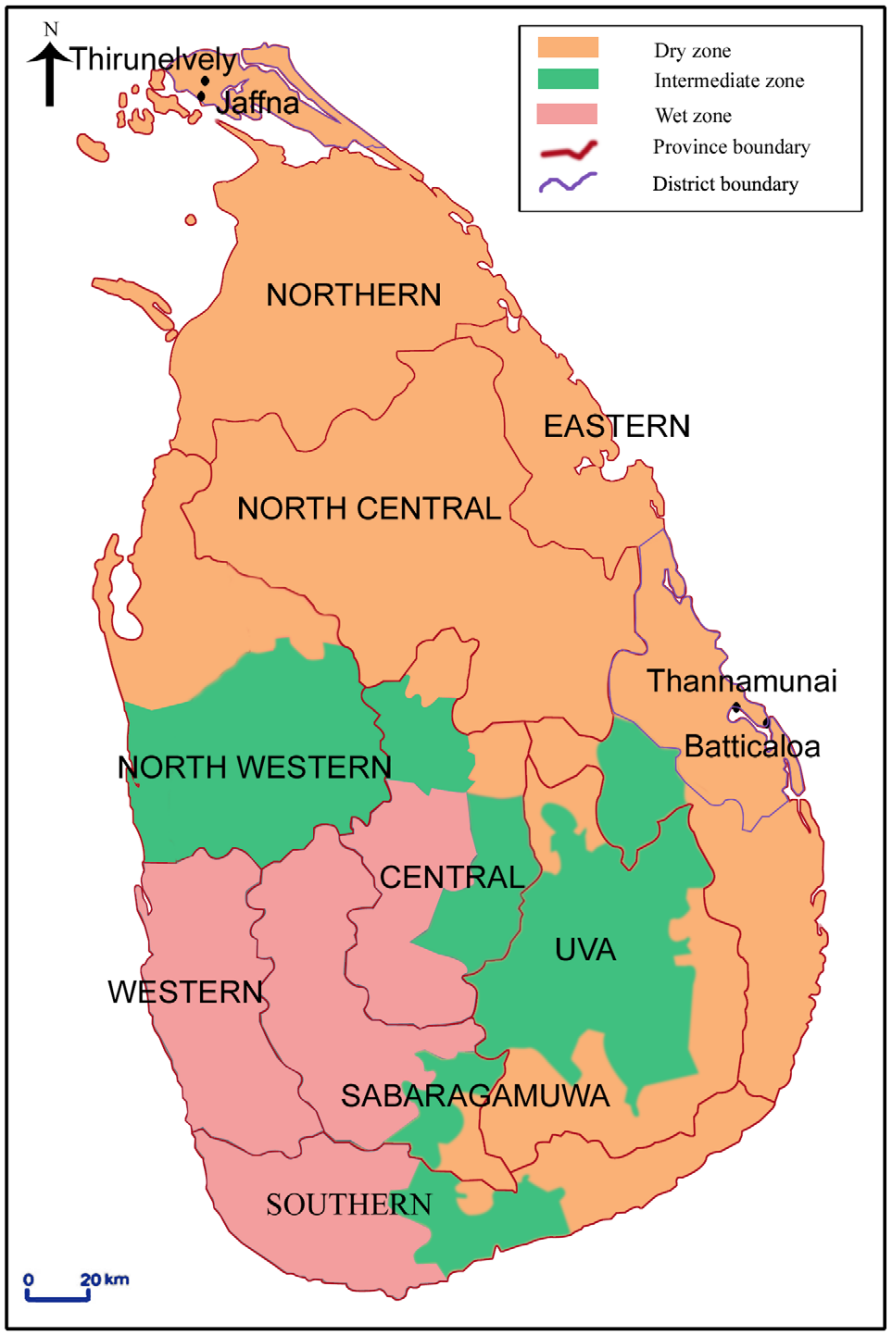
Map showing the locations of study sites in Sri Lanka.

*Aedes* eggs and larvae for experimental studies were collected in Thirunelvely (inland, 9°41′ 03.49″N: 80° 01′ 14.49″E) and Batticaloa town (coastal, 7°43′ 35.81″N: 81° 42′ 4.04″E) in the Sri Lankan districts of Jaffna and Batticaloa respectively. Coastal areas of Jaffna city in the Jaffna district and Thannamunai (7°46′ 06.15″N: 81° 36′ 33.11″E) in the Batticaloa district and specific inland locations in the Jaffna district, were surveyed for larvae in brackish water habitats (Figure 1).

### Collection of *Aedes* eggs and larvae for laboratory experiments

Black plastic ovitraps prepared as described previously [14] were used to collect *Aedes* mosquito eggs and larvae from ten randomly selected houses in Thirunelvely and Batticaloa. Four ovitraps were placed in each house. Each ovitrap, with a capacity of 250 ml, contained 100 ml of tap water and 3×10 cm plywood paddle resting against the upper rim. Ovitrap collections were done every two weeks from August to December 2008. Collected eggs and larvae from Batticaloa and Thirunelvely were brought to the Zoology Laboratories at the Eastern and Jaffna Universities respectively and reared under laboratory conditions (28±2°C, 70–80% R.H.). Larvae along with eggs in each ovitrap were reared separately in yoghurt cups (3.5×6.5 cm) containing 50 ml of tap water. Powdered fish meal pellets were provided twice a day as larval food. The emerging adults were identified using standard keys [16]. Adults identified as *Ae. aegypti* and *Ae. albopictus* were pooled separately and used to establish self-mating colonies that were fed on rabbit blood. The resultant larval progenies were used for subsequent salinity tolerance tests.

### Laboratory experiments on salinity tolerance of larvae

First and early third instar larvae of *Ae. aegypti* and *Ae. albopictus* were exposed to different salinity levels of 0, 2, 4, 6, 8, 10, 12, 14, 16, 18 and 20 ppt in the two laboratories . Required salinities were obtained by adding tap water (<0.5 ppt salinity) to sea water and the salinities measured with a refractor-salinometer (Atago, Japan). Twenty larvae in 150 ml capacity plastic containers containing 100 ml of water of different salinities were maintained at room temperature (28±2°C) until their emergence to adults. Plastic lids were used to partially cover the containers to minimise evaporation. Larvae were fed twice daily with powered fish meal. Three replicate tests were run in parallel for each level of salinity. Although experiments were conducted at different times in the Eastern and Jaffna Universities, the laboratory conditions and procedures used for rearing and testing larvae were the same in the two laboratories. Numbers of adults emerging were determined and the results were recorded as the mean percentage survival of larvae to reach adulthood at each salinity level ± standard errors of the mean.

### Oviposition preference for fresh and brackish water

An ovitrap-based experiment was conducted at Thirunelvely between August and October, 2010 in the dry season to assess the egg laying preferences of *Ae. aegypti* and *Ae. albopictus* for brackish water of varying salinity. Eleven ovitraps with salinities of 0, 2, 4, 6, 8, 10, 12, 14, 16, 18, 20 ppt were prepared by adding tap water to sea water [14] and placed on an aluminum tray. Three similar trays each containing 11 ovitraps were prepared and placed 50 m apart in open buildings with roof cover to prevent dilution in case of rain. Observations were made weekly for six weeks and during each inspection, the larvae in each ovitrap were collected and identified. The solutions in the ovitraps were replaced with fresh solutions after every round of larval collection.

### Brackish water larval development habitats of *Ae. aegypti* and *Ae. albopictus* in Sri Lanka

A larval survey that examined brackish water collections in discarded containers such as tins, glass-bottles, plastic cups and bottles and discarded tyres as well as abandoned boats and disused wells was carried out along the Thannamunai coast from August to October, 2010 and the Jaffna city coast from February to March, 2011. In a parallel study, the salinities of randomly selected domestic wells located along a 6 km stretch of the main road from the Jaffna coast to Thirunelvely were determined and the water examined for *Aedes* larvae. Water samples, with or without *Aedes* larvae, were taken to the Zoology Laboratories of the Eastern and Jaffna Universities for measuring salinity and identifying larvae.

### Dengue incidence in Jaffna city

Data on the number of new dengue cases in different administrative divisions of Jaffna city were obtained from the Medical Office for Health, Ministry of Health, Jaffna for the seven month period from 1 October 2010 to 30 April 2011. Population data for the divisions of Jaffna city were obtained from the Office of the Divisional Secretariat of Nallur and Jaffna. Incidence was calculated as the number of dengue cases per 1000 persons for the seven month period.

### Statistical analysis

The level of salinity producing 50% failure to emerge as adults (LC_50_) and its 95% confidence limits were determined by Probit analysis using Minitab statistical software (Minitab Inc, PA, USA) for each larval population together with LC_50_ ratio tests to additionally determine the significance of LC_50_ variations among the populations as described by Wheeler et al. [17]. Multiple regression analysis was performed on the oviposition data using the Minitab statistical software to determine the relationships between experimental variables. The numbers of larvae of each species collected weekly was considered as the dependent variable and salinity, time and mosquito species as independent variables. Mosquito species were considered dummy variables and ascribed values of 0 for *Ae. aegypti* and 1 for *Ae. albopictus* in the analysis. The multiple regression model therefore had a mixture of quantitative (time and salinity) and qualitative (mosquito species) predictors.

## Results

**Figure 2:**
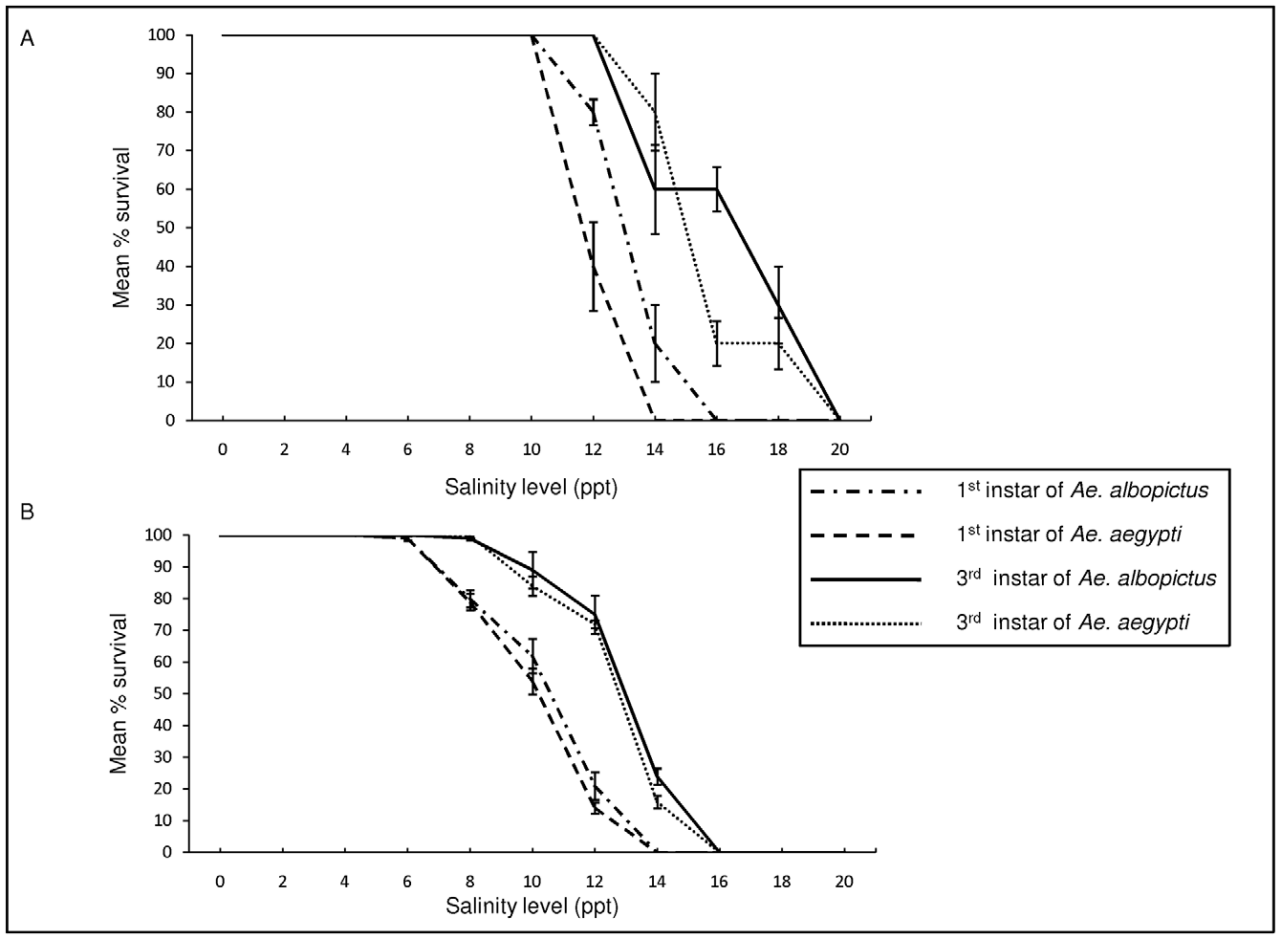
Salinity tolerance of first and third instar larvae of *Ae. aegypti* and *Ae. albopictus*. The results show the salinity tolerance of larvae derived from mosquitoes in the towns of (A) Thirunelvely and (B) Batticaloa, in Sri Lanka. Numbers of adults emerging from larvae were determined in triplicate at varying salinities, and the results were recorded as the mean percentage survival of larvae to reach adulthood at each salinity level ± standard errors of the mean.

The salinity tolerance of *Ae. aegypti* and *Ae. albopictus* larvae originating from Thirunelvely and Batticaloa town in the districts of Jaffna and Batticaloa respectively in Sri Lanka (Figure 1) were initially determined in the laboratory. The results are presented graphically in Figure 2 with relevant parameters tabulated in Table 1. The results show that, in all instances where 95% confidence intervals could be determined for the LC_50_ values, the third instar larvae of both species were significantly more tolerant of salinity at p<0.05 , based on non-overlapping confidence intervals, than the corresponding first instar larvae at both locations (Table 1). These findings were confirmed by LC_50_ ratio tests [17] at the p<0.01 level of significance (Table S1). The non-overlapping 95% confidence intervals (Table 1), confirmed by LC_50_ ratio tests at p<0.01 (Table S1), also suggest that *Aedes* populations in Thirunelvely may be more tolerant of salinity than the corresponding ones from Batticaloa, with the caveat that the experimental comparison was done at different times in two separate laboratories, albeit under similar assay conditions. *Ae. albopictus* larvae also tended to have higher LC_50_ values than the corresponding *Ae. aegypti* larvae at both locations but the differences were not statistically significant by either the 95% confidence interval (Table 1) or LC_50_ ratio (Table S1) tests.

**Table 1:** Salinity tolerance of *Aedes aegypti* and *Aedes albopictus* larvae in the laboratory.

Location	Species	Larval stage	Maximum salinity tolerated for 100% survival to adulthood in ppt	LC_50_ ppt (CI)
Thirunelvely	*Ae. aegypti*	1st	10	11.9 (nd)
	*Ae. aegypti*	3rd	12	15.5 (14.6,16.4)
	*Ae. albopictus*	1st	10	13.0 (12.3,13.7)
	*Ae. albopictus*	3rd	12	16.0 (15.0,17.0)
Batticaloa	*Ae. aegypti*	1st	4	9.8 (8.9,10.6)
	*Ae. aegypti*	3rd	8	12.6 (11.7,13.4)
	*Ae. albopictus*	1st	4	10.2 (9.4,11.1)
	*Ae. albopictus*	3rd	6	12.9 (12.1,13.8)

CI – 95% confidence interval; nd – could not be determined.

The capacity of the two *Aedes* species to lay eggs in field ovitraps containing water with salinity varying from 0 to 20 ppt was also determined. Observations were made weekly for six weeks in Thirunelvely. *Ae. aegypti* and *Ae. albopictus* laid significant numbers of eggs in ovitraps with salinity up to 18 ppt and 16 ppt respectively (Table 2). The analysis of variance showed that the multiple regression analysis model was highly significant (F = 13.9, df = 3, p<0.001) with partial regression coefficients demonstrating that this was due to the numbers of larvae collected per week increasing over time (coefficient = 0.17, T = 2.4, p = 0.016) and decreasing with increasing salinity in the ovitraps (coefficient = −0.77, T = −5.8, p<0.001). However the multiple regression analysis showed that the numbers of larvae collected per week were not significantly different between the two mosquito species (coefficient = −2.49, T = −1.5, p = 0.145).

**Table 2:** Characteristics of *Aedes* oviposition in field ovitraps.

	1^st^ week		2^nd^ week		3^rd^ week		4^th^ week		5^th^ week		6^th^ week		Percent of Total	
Salinity (ppt)	*Ae. aegypti*	*Ae. albopictus*	*Ae. aegypti*	*Ae. albopictus*	*Ae. aegypti*	*Ae. albopictus*	*Ae. aegypti*	*Ae. albopictus*	*Ae. aegypti*	*Ae. albopictus*	*Ae. aegypti*	*Ae. albopictus*	*Ae. aegypti*	*Ae. albopictus*
0	0	26	40	34	14	22	0	26	28	15	23	17	**18.1**	**33.7**
2	0	0	16	0	0	0	22	0	18	0	35	30	**15.7**	**7.2**
4	12	54	30	0	0	4	0	12	15	0	22	0	**13.6**	**16.9**
6	0	0	20	0	0	20	10	0	16	0	6	17	**9.0**	**8.9**
8	5	0	0	0	13	0	34	9	0	0	28	0	**13.8**	**2.2**
10	0	0	19	5	18	0	24	0	0	34	4	18	**11.2**	**13.7**
12	0	0	6	0	7	5	23	0	0	0	15	4	**8.8**	**2.2**
14	0	0	0	0	0	0	8	12	0	13	12	11	**3.5**	**8.7**
16	0	0	0	0	0	0	0	9	10	18	8	0	**3.1**	**6.5**
18	0	0	0	0	0	0	0	0	0	0	18	0	**3.1**	**0**
20	0	0	0	0	0	0	0	0	0	0	0	0	**0**	**0**

The table shows the numbers and the percentage of the total numbers of *Ae. aegypti* and *Ae. albopictus* larvae collected from ovitraps of varying salinities over a six week period.

**Figure 3:**
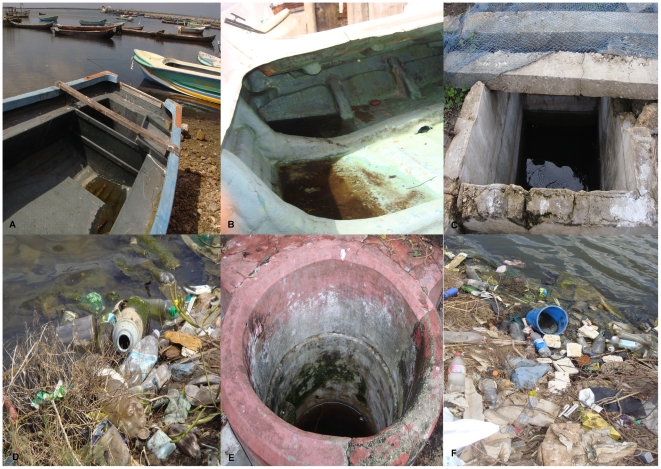
Brackish water development habitats of *Aedes* larvae in Sri Lanka. The photographs show the brackish water collections containing larvae in: A & B - disused boats; C & E: abandoned wells; D & F: discarded food and beverage containers.

**Figure 4:**
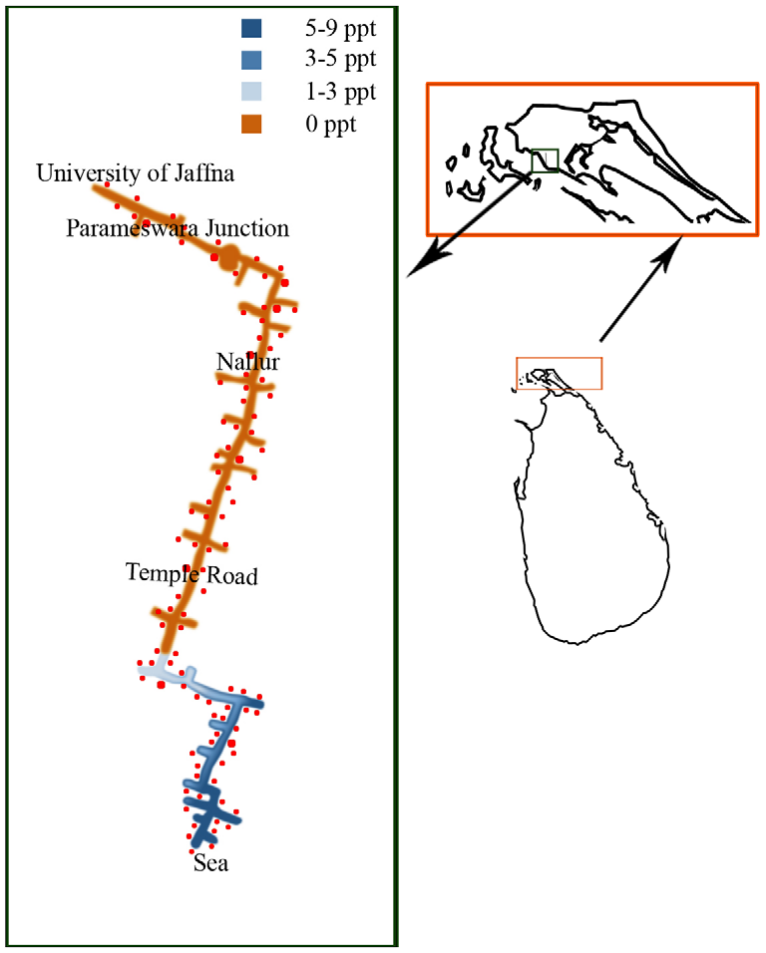
Salinity in domestic wells from the Jaffna coast to Thirunelvely. The results show the changes in salinity in domestic wells that were examined for *Aedes* larvae along a 6 km stretch of the main road from the Jaffna coast to the University of Jaffna campus in Thirunelvely.

Potential brackish water preimaginal development sites of *Ae. aegypti* and *Ae. albopictus* in the coastal peri-urban environment of Thannamunai, Batticaloa district were then investigated over a four month period. Among 83 discarded food and beverage containers with brackish water that were inspected 14 (17%), with salinity levels ranging from 2 to 14 ppt, were found to contain either *Ae. aegypti* or *Ae. albopictus* larvae (Table 3). The salinity of water samples from containers that did not possess *Aedes* larvae varied from 2 to16 ppt. Of 89 potential brackish water habitats subsequently investigated along the coast of Jaffna city, three disused boats and two abandoned wells (6%) were found to contain *Ae. aegypti* larvae in a salinity range 3 to15 ppt (Table 3, with illustrative photographs of habitats in Figure 3). The salinity of brackish water samples from habitats in Jaffna city that did not contain *Aedes* larvae varied from 1–18 ppt. Along the main road from the Jaffna coast to Thirunelvely, 102 frequently used domestic wells were also examined. *Aedes* larvae were not found in such wells, where the salinities ranged from 9 ppt near the sea to 0 ppt in Thirunelvely (Figure 4).

**Table 3:** Brackish water habitats with *Aedes* larvae in Thannamunai and Jaffna.

Locality	Type of site	Location or distance from sea (m)	Salinity (ppt)	Species present	Number of larvae per container/dipper
Thannamunai	Ice cream cup	Lagoon edge	8	*Ae. albopictus*	6
	Jam jar	Lagoon edge	14	*Ae. albopictus*	11
	Ice cream cup	50 m	5	*Ae. aegypti*	7
	Fish tin	Lagoon edge	6	*Ae. albopictus*	9
	Yoghurt cup	10 m	7	*Ae. aegypti*	12
	Glass bottle	Lagoon edge	8	*Ae. aegypti*	27
	Yoghurt cup	100 m	2	*Ae. albopictus*	18
	Fish tin	Lagoon edge	14	*Ae. albopictus*	3
	Jam jar	250 m	7	*Ae. albopictus*	14
	Milk carton	400 m	3	*Ae. aegypti*	21
	Ice cream cup	Lagoon edge	5	*Ae. albopictus*	7
	Ice cream cup	100 m	6	*Ae. aegypti*	4
	Milk carton	Lagoon edge	3	*Ae. albopictus*	18
	Jam jar	15 m	2	*Ae. aegypti*	15
Jaffna coast	Well	100 m	3	*Ae. aegypti*	9 (per 250 ml capacity dipper)
	Well	40 m	5	*Ae. aegypti*	7 (per 250 ml capacity dipper)
	Boat	beach	10	*Ae. aegypti*	14 per boat
	Boat	beach	12	*Ae. aegypti*	6 per boat
	Boat	200 m	15	*Ae. aegypti*	8 per boat

The table shows details of brackish water habitats containing *Ae. aegypti* and *Ae. albopictus* larvae studied in the Batticaloa district from August 2010 to November 2010 and the Jaffna coast from February 2011 to March 2011.

**Figure 5:**
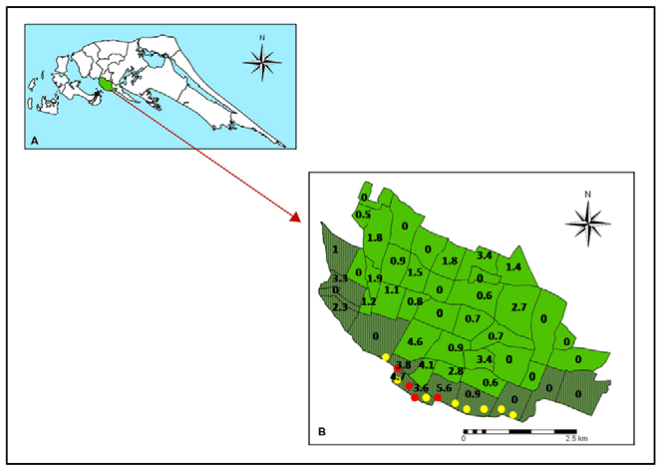
Relationship between dengue incidence and brackish water sites with larvae of *Ae. aegypti* in Jaffna. A - Map of Jaffna peninsula; B – Map of Jaffna city showing its administrative divisions with the coastal divisions shaded in dark green. The numbers indicate the incidence of dengue per 1000 persons for the seven months October 2010 to April 2011 in each division. Red and yellow filled circles show brackish water sites along the Jaffna coastal area that were respectively positive and negative for *Ae. aegypti* larvae. Each circle had one or more container, well or boat that was sampled.

The incidence of dengue in the period 1 October 2010 to 30 April 2011 in Jaffna city, which included the larval survey period of February and March 2011, was relatively high in divisions close to the coastal brackish water sites where *Ae. aegypti* larvae were detected (Figure 5).

## Discussion

Several mosquito species with salinity-tolerant larvae are vectors of human arboviral and parasitic diseases in many parts of the world [18]. They include some members of the genus *Aedes*, e.g. *Aedes togoi* Theobald found in coastal marshes and *Aedes taeniorhyncus* Wiedemann in splash pools, whose osmoregulatory mechanisms have been well studied [19]. *Ae. aegypti* and *Ae. albopictus* in contrast have been widely regarded to undergo preimaginal development only in freshwater [3], [5]. However early laboratory studies showed that *Ae. aegypti* larvae can tolerate limited salinity changes through an osmoconformation mechanism involving the accumulation of amino acids and ions in the haemolymph [20]. Our findings show that *Ae. aegypti* and *Ae. albopictus* can oviposit and undergo preimaginal development in fresh (tap) and brackish waters under field conditions, but that oviposition diminishes with increasing salinity of the water. Previous laboratory studies of oviposition by established laboratory colonies of *Ae. aegypti* showed that it prefers 2.5 ppt salinity compared to distilled water for oviposition [21], [22]. These studies also suggested that *Ae. aegypti* can oviposit in up to 30 ppt salinity under laboratory conditions. However such findings may not be directly applicable to the natural situation where freshwater habitats would contain dissolved minerals that might influence oviposition. They are also not directly comparable to our data with field mosquito populations and tap water from an artesian well source that contains dissolved minerals. A study on the oviposition preference of a laboratory colony of *Ae. albopictus* found that tap water was preferred to 1 ppt and higher concentrations of NaCl [23]. Our data from Thirunelvely suggest that *Ae. aegypti* and *Ae. albopictus* are able to oviposit in a field situation over a relatively wide range of salinity of up to 18 ppt and 16 ppt respectively. This is consistent with the finding of their larvae in brackish water collections of 2 to 15 ppt salinity in Thannamunai and Jaffna city coasts. In the context of the findings in Thirunelvely, it may be relevant to compare the salinity tolerance of the larvae of mosquitoes emerging from fresh and brackish water ovitraps as this can demonstrate potential genetic differences in the field populations of mosquitoes. Water conditioned by previous culture with *Ae. albopictus* larvae enhances oviposition by *Ae. albopictus* [23], presumably due to the presence of chemicals that are sensed by receptors in the gravid female mosquito [22]. This phenomenon could explain the greater oviposition with time in the field ovitraps observed in our investigation. However an increase in mosquito abundance could have also caused or contributed to the increasing oviposition during the six week study period.

*Ae. aegypti* larvae derived from an established laboratory colony showed approximately 80% survival in deionized water, >90% survival at 3.5 ppt salinity, rapid decrease in survival at >8 ppt salinity with an approximate LC_50_ of 14 ppt, and 0% survival at 17.5 ppt salinity in a study by Clarke et al. in the United States [24]. This study further showed that preimaginal development times were prolonged and pupal mass reduced at high salinities [24]. Because of the use of deionized water as the control and for diluting sea water, a different criterion for survival, and the use of larvae from an established laboratory colony in that study, the results are not strictly comparable to our present findings. However, the LC_50_ values for the two *Aedes* species for both first and third instar larvae in Batticaloa tend to be lower and the LC_50_ values for Thirunelvely similar to the LC_50_ observed with the *Ae. aegypti* laboratory colony by Clarke et al. [24]. The greater salinity tolerance of third instar larvae compared to first instar larvae observed in our study may be due to structural and physiological changes related to those that reduce ion permeability in pupae [19].

The Jaffna peninsula in Sri Lanka, unlike Batticaloa, is largely composed of sedimentary limestone and has many lagoons and other inland saline water bodies. Groundwater from aquifers and wells is over-used for domestic and agricultural purposes and therefore groundwater salinization, as illustrated also in Figure 4 for domestic wells, is widespread [25]. This effect is compounded by a high population density of approximately 700 persons per km^2^ in the 1130 km^2^ peninsula. Although Thirunelvely is an area where the accessible ground water in wells is fresh (Figure 4), the small size of the Jaffna peninsula provides many areas with brackish water close enough to Thirunelvely to be within reach of *Aedes* populations. The possibly greater salinity tolerance of the *Aedes* populations in Thirunelvely compared to Batticaloa needs to be confirmed by parallel experiments carried out under identical conditions in the same laboratory but may reflect adaptive genetic differences in the two populations analogous to that seen in *Aedes camptorhynchus* Thomson, a salinity-tolerant vector of Ross River virus in Southwestern Australia [26]. Larvae of coastal marsh populations of *Ae. camptorhynchus* tolerate greater salinity (52 ppt, i.e. hypersalinity) than inland populations (30 ppt, i.e. approaching the average salinity of sea water), probably due to genetic changes in osmoregulatory mechanisms [26]. The gradual adaptation of laboratory colonies of *Ae. taeniorhyncus* to increasing salinity are also likely to be due to genetic changes [19]. Genetic adaptation of mosquitoes to tolerate salinity in preimaginal habitats is also exemplified by differential salinity tolerance among sibling species of *Anopheles* mosquitoes [18].

*Ae. aegypti* and *Ae. albopictus*, are widely adapted to urban and suburban environments in Sri Lanka [27], Brunei [9] and many other countries [1], [10]. Although *Ae. albopictus* is reportedly more exophillic than *Ae. aegypti*, both mosquitoes normally lay eggs within a 1 km radius of their feeding site, thereby ensuring continuing proximity to human hosts [28]. This facilitates the human-mosquito-human transmission cycle which is normal for urban dengue and chikungunya, and during epidemics of yellow fever in urban areas [1]. However sylvatic cycles continue to exist in some regions for yellow fever and chikungunya [1]. Our findings now show that *Ae. aegypti* and *Ae. albopictus* lay eggs and their larvae and pupae survive to emerge as adults in brackish water, and that brackish water habitats with larvae are present in the peri-urban environment in Sri Lanka. The brackish water larval sites identified in this study are located in popular beaches or coastal areas <1 km from densely populated housing, consistent with their potential role in dengue and chikungunya transmission. These findings are compatible with oviposition by the two *Aedes* species in brackish water observed in a field situation, and the extent of salinity tolerance shown by their larvae in laboratory studies. Larval indices like the premises index (PI, the percentage of houses with freshwater containers positive for larvae) are commonly used entomological parameters for assessing the potential for the transmission of dengue by *Ae. aegypti* and *Ae. albopictus* [5], [10]. Dengue outbreaks have occurred in areas with a PI of approximately 2% in Singapore [10] and Cuba [29]. The relevance of 6% and 17% larval positivity rates for brackish water collections in Sri Lanka, although a different measure from the PI, needs to be evaluated further in the context of their potential contribution to the transmission of dengue and chikungunya. The present findings are also consistent with the possibility that sites where larvae of *Ae. aegypti* were found in brackish water may be located in the vicinity of neighborhoods with a higher dengue incidence in the coastal areas of Jaffna city, Sri Lanka. *Ae. albopictus* larvae were also found in brackish water within discarded food and beverage containers, with salinities up to 8 ppt, along the urban coast of dengue-endemic Brunei (R.R., S.N.S., Idris, F., Yasin, K.M., unpublished data), suggesting that our findings in Sri Lanka may be applicable to many other countries. Detailed epidemiological studies are however needed to conclusively demonstrate that preimaginal development of the two *Aedes* species in brackish water contributes to the transmission of dengue, chikungunya and other arboviral diseases.

It is possible that adaptation of the two *Aedes* populations to salinity may be accompanied by alterations in their vectorial capacity and this merits further investigation. The viability of the preimaginal stages, preimaginal development times and fitness of the emergent adults in brackish water habitats in the environment also need to be determined. Our results further suggest that brackish and freshwater domestic wells that are in frequent use are not common habitats for the preimaginal development of the two *Aedes* species, possibly because of regular disturbance of the water surface and rapid water turnover as well as a paucity of decaying organic matter that provide oviposition cues [30].

Small tropical islands and Southeast Asian countries have a high coastline to land mass ratio and therefore proportionately more potential coastal brackish water sites where the two *Aedes* species may undergo preimaginal development. Because of the exclusive focus of larval control measures on freshwater habitats [5], [10], [27], it is possible that *Aedes* vectors undergoing preimaginal development in brackish water collections in artificial containers have unknowingly contributed to the persistence or emergence of dengue and chikungunya in Sri Lanka [7], [8], chikungunya in Reunion [31], and dengue, despite intensive control programs, in countries like Cuba [32], Singapore [10] and Brunei [9]. High and increasing population densities in coastal areas with attendant socio-economic changes [18] and failing refuse collection systems may exacerbate this situation in resource-poor countries. Salinity-tolerant *Ae. albopictus* will further increase the potential for transmission of dengue and chikungunya in temperate zone countries.

Climate (temperature, rainfall, humidity) change due to global warming can expand the geographical range of vector mosquitoes, extend the disease transmission season, shorten the gonotrophic cycle and reduce the time taken for ingested viruses to develop to infectivity in mosquitoes, thereby increasing the propagation rates of arboviral diseases by *Ae. aegypti* and *Ae. albopictus* [1], [2], [4], [33]–[35]. Furthermore, a rise in sea levels consequent to global warming can increase the extent of natural brackish surface water bodies in coastal areas [36], an effect that can be compounded by higher rates of withdrawal of water from freshwater aquifers in coastal areas by expanding populations [37]. A hypothesis, presented in detail elsewhere [18], suggests that rising sea levels can therefore increase the abundance of salinity-tolerant mosquito vectors and lead to the adaptation of normally freshwater vectors to brackish waters, thereby additionally enhancing transmission of mosquito-borne diseases in coastal areas. The predicted increase in the worldwide population density of coastal areas from 87 persons per km^2^ in the year 2000 to 134 persons per km^2^ in 2050 [38] is also likely to exacerbate the situation by increasing human-vector contact.

Our results show that *Ae. aegypti* and *Ae. albopictus* have successfully adapted to oviposit and undergo preimaginal development in brackish water collections in unused wells and discarded artificial containers of up to 15 ppt salinity in the peri-urban environment. Similar salinity levels occur in parts of natural brackish water bodies like lagoons, estuaries, coastal marshes and tidal pools, as well as ponds, lakes and wells near urbanized coasts. There is no evidence at present for the large scale adaptation of *Ae. aegypti* and *Ae. albopictus* to undergo preimaginal development in natural brackish or saline water bodies. Continuous application of vector control methods solely to freshwater preimaginal development habitats in the urban environment may select for genetic changes favoring the development of *Ae. aegypti* and *Ae. albopictus* in artificial collections of brackish water in coastal urban areas, which in turn, could conceivably also lead to their adaptation to natural brackish water habitats in the future. Such changes could have serious consequences for the health of millions of people in many parts of the world, through a higher incidence of dengue, chikungunya and urban yellow fever as well as other rarer arboviral diseases [1], [4], [13]. The presence of *Ae. aegypti* larvae in disused brackish water wells in Jaffna and the possibly greater salinity tolerance of both *Ae. aegypti* and *Ae. albopictus* larvae in the Jaffna peninsula, where there is more salinization of groundwater compared to Batticaloa [25], may herald the beginning of this adaptive process. We recently showed that *Anopheles culicifacies* Giles, the major vector of malaria in Sri Lanka and an established freshwater species [39], is able to undergo preimaginal development in brackish waters of salinity up to 4 ppt in Sri Lanka [40]. Additionally *Anopheles subpictus* Grassi species B with genetic similarity to *Anopheles sundaicus* Rodenwaldt was demonstrated to be an euryhaline species (ability to tolerate a range of salinities) in Sri Lanka with its larvae being isolated from freshwater and 30 ppt salinity water in a lagoon [41]. Our present findings suggest *Ae. aegypti* and *Ae. albopictus* may have also developed euryhaline-like features, and that further investigations are required to characterize possible genetic changes that may be responsible. National and international health authorities however need to recognize the potential impact on human health of brackish water-adapted *Ae. aegypti*, *Ae. albopictus* and other mosquito vectors that were traditionally considered to be freshwater species, and institute appropriate surveillance and control measures.

## Supporting Information

Table S1 **LC_50_ ratio tests.** Results of LC_50_ ratio tests to determine the significance of the differences in salinity tolerance of different larval populations.
(DOC)
