# Supplementary material for: Larval Development of Aedes aegypti and Aedes albopictus in Peri-Urban Brackish Water and Its Implications for Transmission of Arboviral Diseases
Source: PLoS Negl Trop Dis. 2011 Nov 22;5(11):e1369. doi: 10.1371/journal.pntd.0001369 (PMC3222631; doi:10.1371/journal.pntd.0001369)
Supplement: Table S1 — LC50 ratio tests. Results of LC50 ratio tests to determine the significance of the differences in salinity tolerance of different larval populations. (DOC) [file pntd.0001369.s001.doc]

**Table S1. LC_50_ ratio tests.**

Results of LC_50_ ratio tests to determine the significance of the differences in salinity tolerance of different larval populations.

| **Populations** | **Larval stage** | **LC_50_ ratio test statistic** | **Standard error** | **Test statistic**  **Z** | ***P*** |
| --- | --- | --- | --- | --- | --- |
| Thirunelvely *Ae. albopictus* vs Batticaloa *Ae albopictus* | 1^st^ instar | 0.245 | 0.052 | 4.7 | <0.01 |
| Thirunelvely *Ae. aegypti* vs Batticaloa *Ae. aegypti* | 1^st^ instar | 0.196 | 0.830 | 0.2 | 0.80 |
| Thirunelvely *Ae. albopictus* vs Batticaloa *Ae albopictus* | 3^rd^ instar | 0.213 | 0.047 | 4.5 | <0.01 |
| Thirunelvely *Ae. aegypti* vs Batticaloa *Ae. aegypti* | 3^rd^ instar | 0.208 | 0.045 | 4.7 | <0.01 |
| Thirunelvely *Ae. albopictus* vs Thirunelvely *Ae. aegypti* | 1^st^ instar | 0.087 | 0.830 | 0.1 | 0.92 |
| Thirunelvely *Ae. albopictus* vs Thirunelvely *Ae. aegypti* | 3^rd^ instar | 0.033 | 0.044 | 0.8 | 0.44 |
| Batticaloa *Ae. albopictus* vs Batticaloa *Ae. aegypti* | 1^st^ instar | 0.038 | 0.063 | 0.6 | 0.54 |
| Batticaloa *Ae. albopictus* vs Batticaloa *Ae. aegypti* | 3^rd^ instar | 0.029 | 0.048 | 0.6 | 0.52 |
| Thirunelvely *Ae. albopictus* | 3^rd^ vs 1^st^ instar | 0.208 | 0.042 | 4.9 | <0.01 |
| Thirunelvely *Ae. aegypti* | 3^rd^ vs 1^st^ instar | 0.261 | 0.830 | 0.3 | 0.62 |
| Batticaloa *Ae. albopictus* | 3^rd^ vs 1^st^ instar | 0.240 | 0.056 | 4.3 | <0.01 |
| Batticaloa *Ae. aegypti* | 3^rd^ vs 1st instar | 0.249 | 0.056 | 4.5 | <0.01 |
